# Protocol for brain and ventricle volume comparison after clearing and light-sheet microscopy in mice

**DOI:** 10.1016/j.xpro.2026.104877

**Published:** 2026-10-01

**Authors:** Ana Blas-Medina, Minh Son Phan, Samir Ali-Moussa, Nathalie Spassky, Julien Fernandes, Jean-Yves Tinevez, Aleksandra Deczkowska, Laetitia Travier

**Affiliations:** 1 Brain-Immune Communication Lab, Institut Pasteur, Université Paris Cité, Inserm U1224, 75015 Paris, France; 2 PhD Program, École Doctorale Bio Sorbonne Paris Cité (BioSPC), Université de Paris Cité, 75005 Paris, France; 3 Image Analysis Hub, C2RI Université Paris Cité, 75015 Paris, France; 4 Institut de Biologie de l'École Normale Supérieure (IBENS), École Normale Supérieure, CNRS, INSERM, PSL Research University, 75005 Paris, France; 5 Institut Pasteur, Université Paris Cité, Photonic Bio-Imaging Unit, Centre de Ressources et Recherches Technologiques (UTechS-PBI, C2RT), 75015 Paris, France

**Keywords:** Developmental biology, Health Sciences, Microscopy, Model Organisms, Neuroscience

## Abstract

Analysis of brain and ventricular volumes provides insights into the physiological and pathological progression of brain development and aging, but accessible and cost-effective approaches are lacking. Here, we present a workflow to quantify relative brain and ventricular volumes in unsectioned cleared mouse brains following fluorescent detection of brain tissue and ventricle lining. We describe steps for brain harvesting, clearing, imaging by light-sheet microscopy, image resampling, and ventricle annotation, and comparing brains from adult, neonate, germ-free, and hydrocephalic mouse models.

## Highlights

• Volumetric imaging of unsectioned and cleared mouse brains
• 3D visualization of brain ventricles in adult, neonatal, and hydrocephalic mouse models
• Image resampling for efficient processing of large volumetric datasets
• Annotation-based quantification of brain ventricle volume

## Abstract (editor highlights)

Publisher’s note: Undertaking any experimental protocol requires adherence to local institutional guidelines for laboratory safety and ethics.

## Graphical abstract

**Figure:**
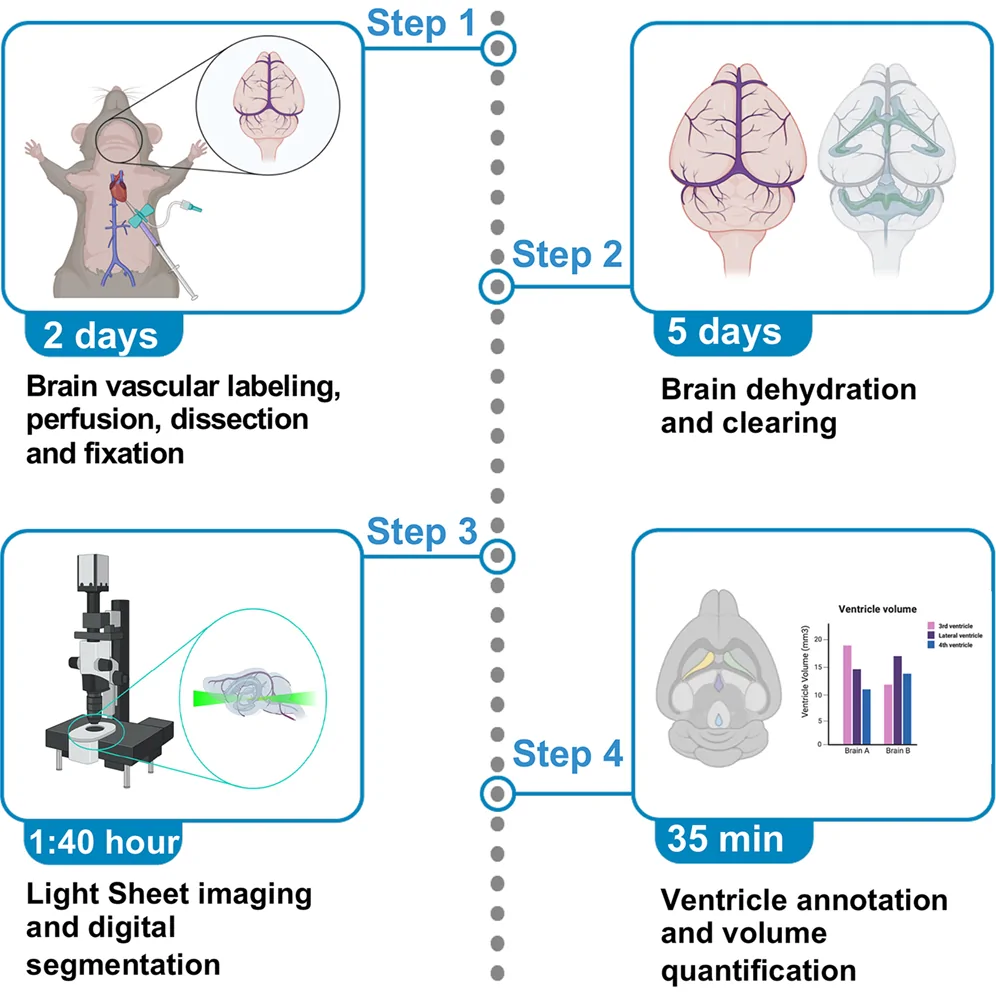

## Before you begin

Brain and brain ventricular volume are key determinants that play an essential role in maintaining the homeostasis of the central nervous system, with the former being determined by the organization of neural and glial cells, and the latter being primarily defined by cerebrospinal fluid flow.^1^^,^^2^ Under physiological conditions, both volumes are stable, reflecting a balance between cerebrospinal fluid dynamics and the surrounding brain parenchyma, but their equilibrium can vary in response to various pathological factors.^3^^,^^4^^,^^5^^,^^6^^,^^7^ In this sense, deviations in brain and ventricular size are recognized as early markers of neurodevelopmental and neurodegenerative disease progression, including autism,^8^^,^^9^ dementia^10^ and hydrocephalus.^7^ Interestingly, such alterations have additionally been identified in mouse models of impaired gut-brain axis communication.^6^

The protocol described below has been developed as an alternative to mouse brain and brain ventricle volume assessment with Magnetic Resonance Imaging (MRI), with the added advantage of enabling reliable analysis of smaller brains, such as neonatal samples, which are often below the practical resolution of conventional MRI.^11^ We describe here a novel workflow based on mouse brain clearing, light sheet microscopy, image processing and ventricle annotation with Arivis Vision4D. We include a detailed procedure of brain ventricle annotation and quantification, based on identification of fluorescently labeled brain tissue boundaries in contrast with the unlabeled ventricular space. To this end, our protocol uses endothelial and nuclear markers that homogeneously label brain tissue, combined with a DISCO-type clearing process that reduces the volume of the brain and allows it to be imaged in three-dimensions using light-sheet microscopy. This protocol does not allow for the measurement of brain and ventricular volumes before clearing and shrinking, but it does enable the detection and measurement of relative changes in various models of adult and neonatal mice. As such, the proposed protocol has been verified in a genetic mouse model of hydrocephalus in neonatal stages^7^ as well as in adult conventional and germ-free mice, which are devoid of a microbiome and have been shown to exhibit a reduced brain volume when compared to their conventional counterparts.^6^ Additionally, this protocol can be adapted for any other disease context in which brain or brain ventricle alterations have been described.

### Innovation

Although MRI can accurately quantify brain and ventricular volumes, it remains inaccessible for many research settings, and cost-effective alternatives based on two-dimensional quantification often fail to capture true ventricular size.^12^ The proposed protocol overcomes these limitations by providing an accessible and reliable workflow for assessing three-dimensional volume of the brain, while being adaptable to different experimental models and tissue clearing strategies. Our methodology relies on equipment and reagents commonly available in neuroscience laboratories, including Python scripts for image processing and Arivis Vision4D software for ventricle volume annotation, and has been validated in adult and neonatal mouse models of health and disease. In addition, the approach enables efficient processing of numerous samples with high resolution and short scanning times. Thereby, the proposed workflow fills a methodological gap for researchers studying neurodegeneration, hydrocephalus and especially neurodevelopment, a field where the small size of the newborn brain is a common constraint in image resolution.^11^ Overall, the protocol will facilitate the assessment of variations in brain and ventricular volume and could therefore be used to identify early signs of neurodevelopmental and neurodegenerative disease onset.

### Institutional permissions

All the procedures on mice agree with the guidelines of the European Commission for the handling of laboratory animals, Directive 86/609/EEC, approved by the ethical committees No. 59 by the CETEA/CEEA No.089 and No. CEE1-005 Charles Darwin, under the numbers dap9343, dap44876, dap 210067 and APAFIS #32382-2021070917055505.

Researchers using this protocol must have permission and follow the rules of their local authorities.

## Key resources table

**Table:** 

REAGENT or RESOURCE	SOURCE	IDENTIFIER
**Chemicals, peptides, and recombinant proteins**		

Lycopersicon esculentum (Tomato) Lectin DyLight 488 1mg/ml	Thermo Fisher Scientific	Cat # L32470
TO-PRO-3 Iodide (642/661) 1mM	Thermo Fisher Scientific	Cat # T3605
Triethylamine	Sigma-Aldrich-Merck	Cat # 471283
Tetrahydrofuran anhydrous	Sigma-Aldrich-Merck	Cat # 401757
Dichloromethane	Sigma-Aldrich-Merck	Cat # 270997
Dibenzyl ether	Sigma-Aldrich-Merck	Cat # 33630
Paraformaldehyde 32% Solution	Fisher Scientific	Cat # 473779M
Heparin sodium salt	Sigma-Aldrich-Merck	Cat # H3149-10KU
DPBS with calcium and Magnesium	Fisher Scientific	Cat # 11580456
DPBS w/o calcium and magnesium	Fisher Scientific	Cat # 14190250
Ketamine 1000	Virbac	GTIN # 03597132111010
Xylazine (Rompun) 2%	Elanco	GTIN # 04007221032311
Sterile physiological NaCl 0.9%	Distrimed	Cat # 873202
Cyanoacrylate glue precision 5G	Loctite	Cat #6.514.882

**Deposited data**		

Raw and analyzed data	This paper	BioImage Archive: S-BIAD2844

**Experimental models: Organisms/strains**		

Mouse: C57BL/6J (Males and Females, 8-12 weeks)	Janvierlabs	SC-C57J-F SC-C57J-M
Mouse: Germ-free C57BL/6J (Males and Females, 8-12 weeks)	Axenic-Gnotobiotic Facility, Institut Pasteur	N/A
Mouse: Nestin: Cre;Ift88^fl/ko^ (Males and Females, Postnatal Day 8)	Provided by Nathalie Spassky	Foerster et al.^7^
Mouse: Nestin::Cre;Ift88^fl/+^ (Males and Females, Postnatal Day 8)	Provided by Nathalie Spassky	Foerster et al.^7^

**Software and algorithms**		

ImspectorPro v. 7.5.4	LaVision Biotec	Version 7.5.4
Imaris v. 10.2.0	Bitplane	https://imaris.oxinst.com/versions/10
Fiji v2.16.0	Schindelin et al.^13^	https://imagej.net/software/fiji/
Python (v3.10.19)	Python Software Foundation	https://www.python.org/
Conda (v23.11.0)	Conda-forge	https://github.com/conda-forge/miniforge
Tifffile (v2025.5.10)	Christoph Gohlke	https://github.com/cgohlke/tifffile
NumPy (v2.2.6)	Van der Walt et al.^14^	https://numpy.org/
Pandas (v2.3.3)	Pandas Development Team	https://pandas.pydata.org/
Jupyter notebook (v7.5.0)	Project Jupyter	https://jupyter.org/
Arivis Vision4D (v4.1.2)	Zeiss	https://www.zeiss.com.sg/microscopy/l/campaigns/software/2023/arivis-vision4d.html
Wacom driver version 6.4.11-1	Wacom	https://www.wacom.com/en-us/support/product-support/drivers
GraphPad Prism v10.2.2	GraphPad Software	https://www.graphpad.com/

**Other**		

Forceps	FST	Cat # 11000-13
Fine scissors - toughcut	FST	Cat # 14058-11
LabM1-II peristaltic pump	Shenchen	Cat #YZ1515X
Microscope Lightsheet UltraMicroscope II	Miltenyi	RRID:SCR_027238
Stereomicroscope SMZ745T	Nikon	Cat # SMZ745/745T
Wacom tablet Cintiq 22 DTK-2260	Wacom	Cat # DTK2260K0A
Eppendorf centrifuge tube 15mL	Dutscher	Cat # 934279B
Eppendorf 5ml tube	Dutscher	Cat # 933006
Microlance 3™ sterile hypodermic needle, gauge 23	Dutscher	Cat # 300800

## Materials and equipment

• Anesthetic mixtures

**Table:** 

Reagent	Final concentration	Amount
Ketamine (stock concentration: 100 mg/mL)	15 mg/mL	300 μL
Xylazine (stock concentration: 20 mg/mL)	0.5 mg/mL	50 μL
Saline	1×	1.65 mL
Total	–	2 mL

Prepare immediately before use, stock at 4°C for up to 24 h.

• Vascular staining solution

**Table:** 

Reagent	Final concentration	Amount
*Lycopersicon Esculentum* Lectin DyLight 488	0.5 mg/mL	0.5 mL
PBS with Ca^2+^ and Mg^2+^ (PBS^+/+^)	1×	0.5 mL
**Total**	–	1 mL

Prepare immediately before use and keep on ice. Do not store.

• Heparin solution

Heparin 10U/ml in 1X PBS^+/+^. The solution is stable at 4°C for 35 days.

• Fixation solution

**Table:** 

Reagent	Final concentration	Amount
Paraformaldehyde 32%	4%	12.5 mL
PBS^+/+^	1×	87.5 mL
**Total**	–	100 mL

Prepare immediately before use and do not store.

**CRITICAL:** PFA is hazardous and must be handled inside a chemical hood. Wear a lab coat, protective gloves, clothing and safety glasses, and follow recommendations when using and discarding PFA.

• Dehydration solutions

**Table:** 

Dehydration solution	Final THF concentration	THF amount	Demineralized water amount	Triethylamine amount	Total volume
THF 50%	50% (v/v)	10 mL	10 mL	∗To pH 9.0	20 mL
THF 70%	70% (v/v)	14 mL	6 mL	∗To pH 9.0	20 mL
THF 80%	80% (v/v)	16 mL	4 mL	∗To pH 9.0	20 mL
THF 100%	100% (v/v)	20 mL	0 mL	∗To pH 9.0	20 mL

Prepare immediately before use and do not store.

• Ventricle lining marker

**Table:** 

Reagent	Final concentration	Amount
TO-PRO-3 Iodide (642/661)	1:1000	5 μL
DBE	N/A	4.995 mL
Total	–	5 mL

Prepare immediately before use and do not store.

## Step-by-step method details

### Transcardial perfusion, vascular labeling, and brain harvesting

**Timing: ∼22 h total (∼30 min per adult brain harvest; ∼20 min per neonate brain harvest; 21 h of brain incubation)**

These steps describe the perfusion and fluorescent vascular labeling of the brain, followed by its extraction and fixation to preserve tissue structure for subsequent processing.

1. Anesthetize the animal with preferred anesthetic (e.g., xylazine/ketamine mixture (see materials and equipment Setup section) administered at 10μl/g via intraperitoneal or subcutaneal injection).
  a. Perform a toe pinch to ensure the animal is unresponsive and fully under anesthesia before starting.
    **CRITICAL:** All live animal procedures must comply with institutional animal care and use guidelines.
  b. Place anesthetized animal in the supine position on a styrofoam surface on an aluminum tray.
  c. Secure each paw to the surface with tape or pins to ensure that the animal lies flat and immobile.
2. Expose the heart of the anesthetized animal and perform vascular fluorescent labeling.
  a. Pinch the skin of the abdomen with forceps and make an incision to expose the abdominal cavity.
  b. Open the abdominal cavity, grip the xiphoid and cut the diaphragm with fine scissors.
    i. Make two cuts on both sides of the rib cage.
    ii. Lift the sternum over the head of the mouse to fully expose the heart and lungs.
      ***Note:*** The rib cage can be pinned to the perfusion station for better accessibility.
  c. Inject 5 μl of vascular staining solution (see materials and equipment Setup section) *per* gram of body mouse weight into the left ventricle, corresponding to 2.5 μg of *Lycopersicon esculentum* Lectin DyLight 488 *per* gram of body mouse.
  d. Allow the vascular staining solution to circulate systemically for 2 min before proceeding to the following steps.
    ***Note:*** The quality of vascular labelling depends on the heart rate of the anaesthetized animal, which is reduced but must remain stable. Excessively deep anesthesia and an excessively slow heart rate can reduce the systemic distribution of the fluorescent tracer.
    ***Note:*** Since the brain is a highly vascularized structure, an endothelial fluorescent marker was selected to enable the uniform detection of brain tissue by fluorescence, enhancing its contrast with the unlabeled brain ventricles. Alternative fluorescent markers, specific to the endothelium or other tissue structures, can be compatible with the present protocol. However, it is not recommended to rely on the tissue’s autofluorescence as it results in a lower contrast between the tissue and the ventricular space.
3. Perform transcardiac perfusion and fixation in a chemical hood.
  a. Inject 20μl of heparin solution (see materials and equipment Setup section) for adults and 10μl in neonates into the left ventricle.
  b. Make an incision in the right atrium of the heart using fine scissors to allow for blood outflow.
  c. Start the perfusion pump, insert the needle in the left ventricle and slowly perfuse with 15ml or 30ml (neonates or adults) of PBS^+/+^ (flow rate = 8ml/min for adults and 5.5ml/min for neonates).
  d. After PBS^+/+^ perfusion is complete, perfuse with 15ml or 30ml (neonates or adults) of fixation solution in a chemical hood (see materials and equipment).

***Note:*** If the perfusion with 4% PFA is performed adequately, the body of the perfused mice should be stiff.

**CRITICAL:** For safety and convenience, we recommend performing the entire procedure under a chemical hood, although strictly speaking, all steps not involving PFA can be carried out on a standard laboratory bench.

***Note:*** Brain vasculature can still be slightly visible after perfusion due to the vascular staining solution.

4. Harvest and post-fix the brain.
  a. Decapitate the mouse immediately after transcardiac perfusion.
  b. Use scissors to remove the scalp, making an incision along the midline from the neck to the nose to expose the skull.
  c. Make lateral cuts on the right and left parts of the skull and carefully remove it with forceps to expose the brain.
  d. Use forceps to sever the nerve bundles on the ventral surface of the brain and scoop the organ out.
  e. Perform post-fixation of the brain in a 15ml-tube by immersion in 5ml of fixation solution for 4h, at 24°C.
  f. Rinse the brain three times in 15ml of PBS without calcium and magnesium (PBS^−/−^) with mild agitation at 24°C, followed by a 16-h incubation at 4°C.

**Pause point:** Although it is recommended to proceed with the protocol on the following day to preserve the staining intensity, brains can be kept at 4 °C for a longer period if needed.

### Brain dehydration and clearing

**Timing: 5 days**

These steps describe the process of whole brain clearing following the FDISCO+ protocol.^15^ This clearing method allows preservation of the fluorescent signal (DyLight 488 fluorophore) of the lectin derived from *Lycopersicon esculentum* during clearing, whilst allowing the brain to shrink, thereby enabling its imaging by light-sheet microscopy.

**CRITICAL:** Several chemicals used in this step are hazardous and must be used and disposed with precaution. All clearing steps should be performed in a chemical hood.

5. Dehydration.
  a. Incubate each brain in solutions of increasing concentration of THF at 4°C (see materials and equipment Setup section), without agitation.
    i. Place the sample in 50%, 70% and 80% THF for 12h at each concentration.
    ii. Next, incubate the sample in 100% THF for three consecutive 12-h periods, refreshing the solution between each incubation (Table 1).

**Table 1:** Schematic of the brain dehydration and clearing protocol

Solvent	Incubation time	Temperature
THF 50%	12 h	4°C
THF 70%	12 h	4°C
THF 80%	12 h	4°C
THF 100% (first incubation)	12 h	4°C
THF 100% (second incubation)	12 h	4°C
THF 100% (third incubation)	12 h	4°C
Dichloromethane	20-30 min	24°C
DBE	Until clearance (usually 2-3 days)	24°C

***Note:*** All incubations are performed in 5ml Eppendorf tubes wrapped with aluminum foil to protect the samples from light. Tubes should be filled as much as possible to limit the presence of oxygen.

6. Delipidation.
  a. Transfer the brains into 5ml-tube containing 100% Dichloromethane until it sinks to the bottom of the tube (about 20 to 30min at 24°C) (Table 1).
7. Clearing.
  a. Prepare ventricle lining marker (see materials and equipment Setup section).
    ***Note:*** TO-PRO-3 Iodide (642/661) is generally used as a nuclear marker, but in this study, it appears to serendipitously label the outline of the brain and ventricles. By adjusting the intensity parameters of the excitation/emission laser pair on the light sheet microscope to enable the detection of TO-PRO-3 Iodide (642/661) at the edges of the brain and ventricles, we can no longer detect the signal from the cell nuclei.
  b. Place each brain in a new plastic tube filled to the maximum with ventricle lining marker (Table 1).
  c. Incubate at 24°C without agitation until clearance (usually takes 2-3 days, we recommend doing it over the weekend). See troubleshooting 1.
    ***Note:*** If brain in DBE is not transparent enough, immerse the brain again in 100% THF for 12h and re-do the delipidation and clearing steps (steps 6 and 7).
    ***Note:*** Dehydration steps in FDISCO clearing protocol shrink brains about 30% (Figure 1). The dehydration process may vary slightly in quality from one experiment to another. To avoid batch effects, we recommend including all brains from the experimental groups to be compared in a single experiment. To control for potential differential brain shrinkage between conditions, it is recommended to measure the organs before and after clearing.
    **Pause point:** The sample can be stored in DBE at 24°C until imaging (always protected from light). Samples can be stored for months while preserving their fluorescence intensity, but it is advisable to proceed to image acquisition in the weeks following clearing.

**Figure 1:**
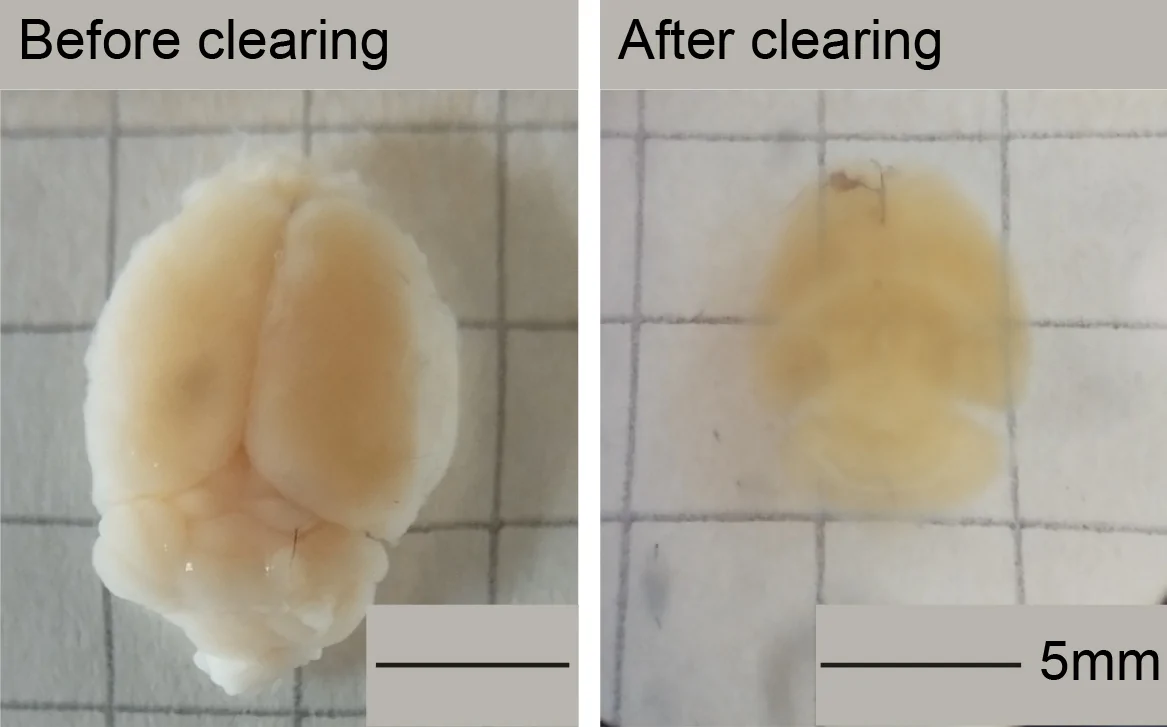
Mouse adult brain before (left) and after (right) clearing process Square size: 5 mm × 5 mm. The olfactive bulbs are removed to favor brain stability when mounted in the imaging holder.

### Light-sheet imaging of cleared brains

**Timing: 1 h per brain**

These steps describe the process of image acquisition for cleared brains on the Miltenyi Ultramicroscope II Light Sheet Microscope and the ImspectorPro software.

***Note:*** This protocol is described with a commercial light-sheet microscope but can be performed equally well with open-source Light Sheet Fluorescent Microscopy systems.

8. Fill the imaging cuvette with DBE until there is enough liquid to cover a full brain.

**CRITICAL:** Always use a dipping cap when handling the light sheet objective to avoid damaging the lens. Handling DBE should be done with care, using a fume hood is recommended. Ethyl Cinnamate can be used as an alternative to DBE.

9. Decide on the optimal orientation and mounting method for brain ventricle imaging, which should be kept consistent across samples.
  a. Place the brain so that the light sheet passes through the width of the brain rather than the length, ensuring that light sheet propagation is as short as possible.
    ***Note:*** Even though the tissue is cleared, longer propagation paths of the light sheet may induce higher light diffraction, which can decrease fluorescence excitation.
  b. Position the brain with the dorsal side facing up (as in its natural anatomical orientation), to ensure that the greatest part of the ventricular system is located at the top.
    ***Note:*** Samples should always be mounted with the region of interest positioned as close to the objective as possible, reducing the distance that the emitted light must travel through the tissue and thereby enhancing image quality. Since the ventricular system is found throughout the brain, with the third and lateral ventricles located more dorsally and the fourth ventricle positioned more ventrally, there is no optimal orientation that ensures equal image quality for all ventricles. The current protocol prioritizes image quality in the dorsal ventricles, whose volume changes are more consistently associated with hydrocephalus and ventriculomegaly,^16^ although a change to an inverted orientation can be considered when studying the fourth ventricle.
  c. Choose a sample holder that will allow for the imaging of the whole brain.^17^
    ***Note:*** When using a holder with a screw, position the brain so that it fits entirely within the vertical walls of the holder, in such a way that its dorsal surface does not rise above the holder’s vertical boundaries.
    ***Note:*** A flat holder without borders can also be used, immobilizing the brain with glue. While the lateral sides of the brain will not be blocked, the glue will stick to the sample, and it cannot be removed completely. The glue can increase the tissue autofluorescence and decrease the quality of the imaging.
    ***Note:*** For samples that are difficult to mount with screw holders or glue, embedding in 1% low-melting agarose can facilitate handling. It is important to embed the tissue before starting the clearing protocol so that both the sample and the agarose undergo the same protocol.
10. Gently mount the brain in the sample holder in the desired orientation. Position the organ as flat as possible (Figure 2A). See troubleshooting 2.

**Figure 2:**
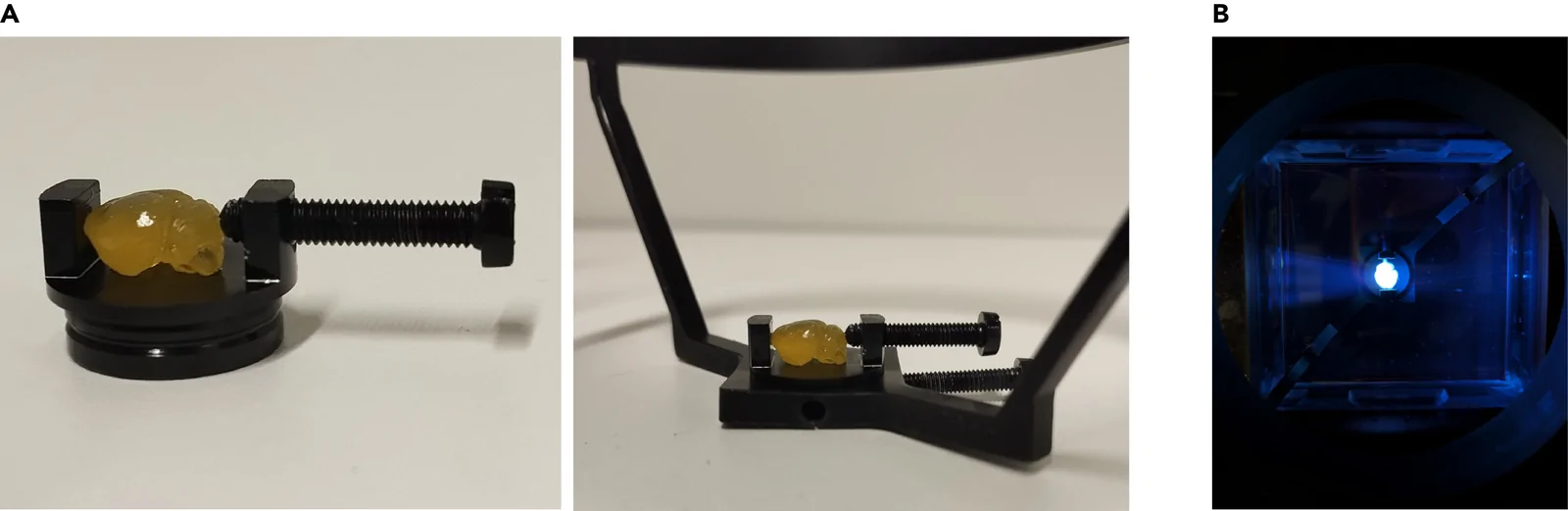
Preparation of cleared brain prior to light sheet imaging (A) Cleared adult mouse brain mounted in the imaging holder and stabilized with a screw. (B) Adjustment of the position of the mounted cleared brain in the DBE cuvette to allow its illumination.

***Note:*** Be mindful of the pressure applied while immobilizing the sample, as excessive force may cause tissue rupture, but insufficient stabilization can result in sample movement or flotation upon immersion in the DBE cuvette.

***Note:*** Olfactive bulbs can be removed to favor the stability of the brain in the holder.

11. Position the sample and adjust the illumination settings.
  a. Lower the brain in the sample holder into the cuvette of the microscope.
  b. Switch on one of the visible lasers in the “Ultrall/Channel Setup” with low laser power (10% selected in the “Ultrall/Laser Power” section) and move the sample in the X and Y directions until the desired position (Figure 2B).
  c. Adjust the sample position in the Z direction so that the light sheet illuminates the top of the tissue before immersing the objective.

***Note:*** This will prevent the objective from crashing into the sample during lowering.

12. Set the correct settings for lateral and third ventricle imaging (Figure 3A).
  a. Set up the adequate objective zoom allowing visualization of the entire brain on the microscope and apply it in the “Ultrall/Objective/Zoom” section on the imaging software.
    ***Note:*** With a 2X objective, zoom is set up at 0.63 to visualize an entire adult brain.
  b. Select the filter couple (excitation/emission) corresponding to the 488nm fluorophore in the “Ultrall/Channel Setup” section to detect the signal from the endothelial marker *Lycopersicon esculentum* Lectin DyLight 488.
  c. Adjust it at a low power (10%) in the “Ultrall/Laser Power” section and switch it on with the main button in the “Measurement Wizard”.
    ***Note:*** In this protocol, fluorescent markers that label the vascular structure and cell nuclei have been selected to enable uniform fluorescence detection of brain tissue and the detection of ventricles by contrast. If other fluorescent markers are used, the user must adjust the excitation/emission laser pair accordingly. If no fluorescent markers have been used, the excitation/emission laser pair offering the highest tissue autofluorescence should be selected.
  d. Lower the objective into the DBE cuvette.
  e. Once inside the liquid, use the mix-max intensity function to visualize the brain and progressively adjust the focus while slowly descending the objective.
    ***Note:*** This function adjusts the limits of the colormap scaling so that the lowest-intensity pixel in the displayed image is set as the lower limit of the colormap and the highest-intensity pixel defines the upper limit, respectively.
  f. Select the focus to create a well-defined image of the lateral and third ventricles.
  g. Adjust the width of the light sheet in the “Ultrall/Optic/sheet width” section and the illumination strategy in the “Ultrall/Lightsheet” section to illuminate the whole width of the brain.
    ***Note:*** Dual-sided illumination and 30% light sheet width are recommended (Figures 3B–3D). See troubleshooting 3.
    ***Note:*** One-sided illumination can be used to reduce imaging time per brain, and it would still be possible to identify the whole ventricular system, however, there are notable intensity differences between the ventricle located closer to the light beam and that located in the contralateral side of the brain.
  h. Click on “Camera” in the “View” menu to set the exposure time.
    ***Note:*** Keep exposure time low to minimize imaging time (e.g. 100ms).
  i. In the “UltraII” menu, adjust the laser power for imaging to the minimum required to visualize the signal while avoiding photobleaching.
  j. Repeat step i for the filter couple that allows detection of the TO-PRO-3 Iodide (642/661) signal.
  k. Scan the whole tissue in the Z direction with both lasers to check that all the parameters have been set correctly.

**Figure 3:**
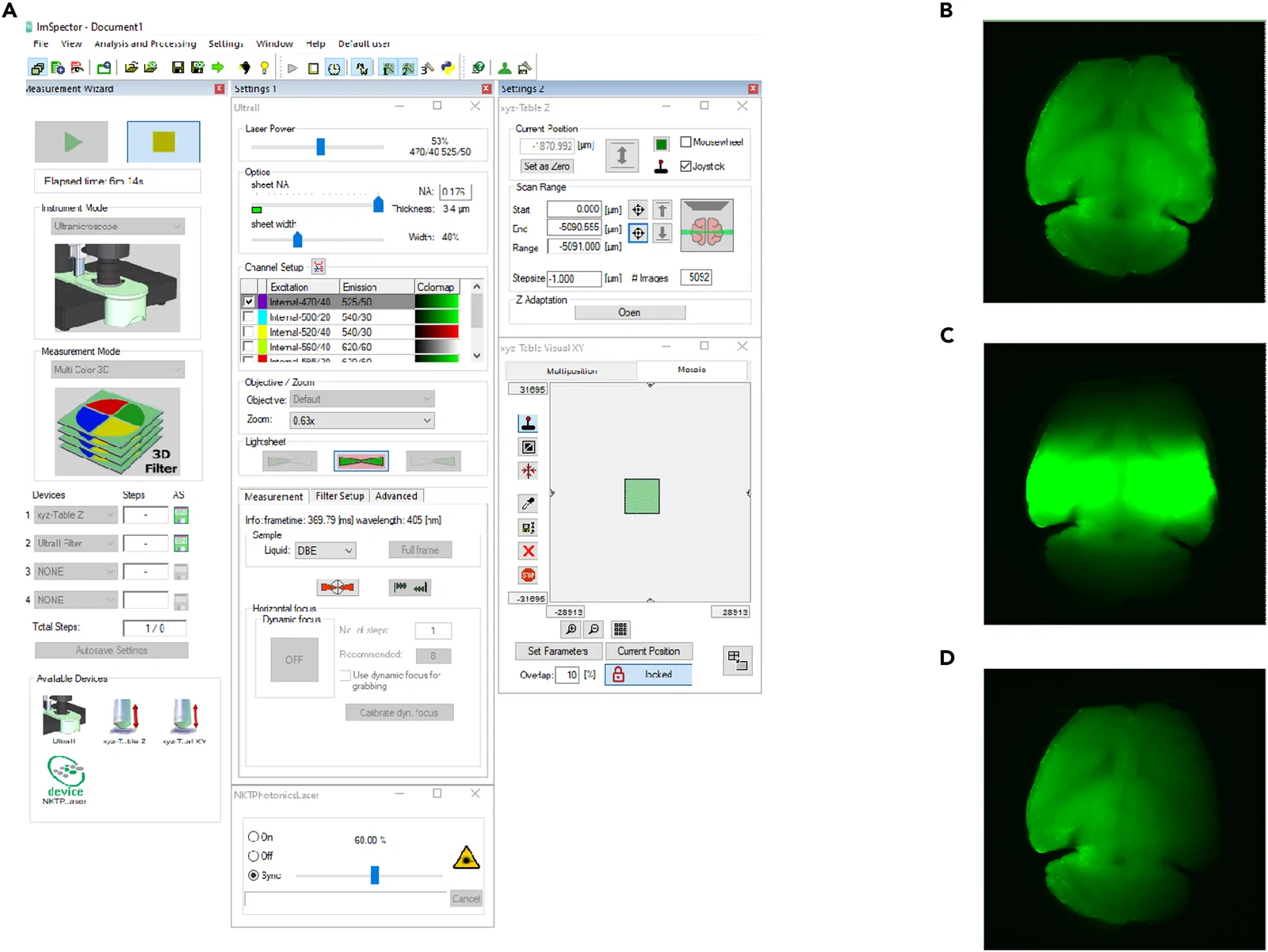
Configuration of the light sheet imaging parameters to image cleared brains stained with *Lycopersicon esculentum* Lectin DyLight 488 (A) View of the different parameters on the ImspectorPro software related to the Miltenyi Ultramicroscope II Light Sheet Microscope. (B) Dual-sided illumination of the brain with 30% light sheet width allowing homogeneous detection of the signal in the X and Y dimensions. (C) Dual-sided illumination of the brain with only 10% light sheet width induces heterogenous signal detection in the Y dimension. (D) One-sided illumination (left sided) of the brain with 30% light sheet width allows greater detection of the signal on one side of the brain.
13. Set up the acquisition range in the Z dimension.
  a. Switch back to the 488 nm illumination parameters.
  b. Without moving the objective, move the sample in Z until the most superficial visible plane of the brain, designate it as Z=0 in the software selecting “Set at Zero” in the “x-y-z Table Z/Current position” section.
  c. Select “Start” in the “x-y-z Table Z/Scan Range” section.
    ***Note:*** The “Set at Zero” selection prevents excessive movement in Z direction while inspecting the sample.
  d. Without moving the objective, move the sample in the Z direction until the deepest illuminated plane of the brain is reached, and designate it as the final Z position selecting “End” in the “x-y-z Table Z/Scan Range” section.
    ***Note:*** After defining the range of acquisition, the user should verify whether the selected range falls within the limit of the microscope’s working distance (4mm for the Miltenyi Ultramicroscope II). In some cases, adult brain samples can be larger than the working distance and the objective will contact the brain as it is reaching the lowest Z position. If this is the case, the final Z position will correspond to the last plane that can be imaged before the objective touches the sample. See troubleshooting 4.
  e. Check the selected range in Z with the 647nm illumination parameters.
14. Set the step size to 1 μm in the “x-y-z Table Z/Stepsize” section.
15. Autosave the scan and start acquisition.

***Note:*** With the step size of 1 μm, the acquisition file corresponds to a folder containing as many.tiff images as the defined range per selected laser. The size of the folder containing the images is about 60 GB for one color laser.

***Note:*** The voxel size that will be required in step 20 is saved in the metadata of the first image of the folder.

***Note:*** Acquired brain images can be imported into Imaris Viewer software, which creates a unique.ims file that is smaller in size than.tiff files, making it easier to process and visualize images in two or three dimensions.

16. If the brain is larger than the microscope’s working distance, adjust the objective after the acquisition and measure the full brain height. See troubleshooting 4.
  a. Slightly lift the objective to a new position where it no longer contacts the sample, but the full brain remains visible, even if blurry.
  b. Without moving the objective, scan the whole tissue in the Z direction to ensure that the most dorsal and ventral planes of the brain are visible.
  c. Redo steps 13b-13d to obtain the full size of the brain in the Z direction.
  d. Record this value for downstream brain volume quantification (see step 33).

### Image resampling

**Timing: 40 min per brain**

These steps resample the image stack obtained in the Light Sheet Microscope to reduce its size by dividing the whole image stack into groups of substacks with a fixed number of z planes. Then, maximum intensity projection is applied for each substack to produce a resampled plane.

**CRITICAL:** Resampling of the image stack uses the Python code provided in the jupyter notebook “z_projection.ipynb”. The jupyter notebook is found at https://gitlab.pasteur.fr/iah-public/ventricle-volume-measurement. Users must follow the instruction in “README.md” to install the Python packages required for running the notebook. The notebook is organized in a series of groups of code, and each group of code is called a cell. The user must run the code iteratively cell by cell from the beginning to the end of the notebook. The input dataset must be organized as a series of images corresponding to specific channel indices and Z planes (step 15).

17. Divide the image stack into groups of substacks by running the jupyter notebook “z_projection.ipynb”.
  a. Run Step 1.1 of the jupyter notebook “z_projection.ipynb” to set up the resampling parameters. Specify:
    i. “DATASET” as path to the dataset.
    ii. “OUTDIR” as path to save the resampled images.
    iii. “NUM_CHANNELS” as the number of channels imaged on the Light Sheet microscope.
    iv. “(Z_BEGIN, Z_END)” as the top and the bottom z planes from which the resampling is computed.
      ***Note:*** If the scan range has been set correctly in the Light Sheet microscope (step 13), “Z_BEGIN” and “Z_END” should correspond to the first and last images of the dataset, representing the most dorsal and most ventral plane of the brain, respectively.
      ***Optional:*** If the user is unsure about which planes to select for the “Z_BEGIN” and “Z_END” parameters, a pre-inspection of the stack can be done in Fiji/Image J software. For this, the “use virtual stack” option is recommended.
    v. “Z_RATIO” as the proportion of the number of Z planes in each substack.
      ***Note:*** The default recommended value in this workflow corresponds to 1.6% of the total number of Z planes. This ratio is recommended to obtain a small enough number of substacks for manual annotation while still maintaining the continuity and smoothness of the structure across the depth in z dimension. For example, for the voxel size of 5.16 x 5.16 x 1 μm (xyz) before resampling, the voxel size after resampling is of 5.16 x 5.16 x 49μm (xyz), allowing annotation with a precision of 50μm to annotate ventricles edges on the entire brain.
      ***Note:*** The degree of compression is = (1 – (1 / (alpha ∗ N))), where alpha is the proportion of z used for grouping (1.6% in this case) and N is the total number of z planes. Example: if N = 3400 planes and alpha = 1.6%, so the compression achieved in Z is about 98.
    vi. “Z_STEP” as the sampling step in Z. This should correspond to the stepsize selected in step 14.
    vii. “FILE_PATTERN” as the file name pattern of the images autosaved in the Light Sheet microscope.
      # Path to the input dataset
      # Use '/' for Linux or Mac system, '\\' for Windows
      > DATASET = "..\\..\\.."
      # Path of the output directory for saving the results
      # Use '/' for Linux or Mac system, '\\' for Windows
      > OUTDIR = "..\\..\\.."
      > NUM_CHANNELS = 2 *# number of channels*
      # The z begin and end from which we compute the downsampling
      > Z_BEGIN = 2 *# z_begin must be >= 1.*
      > Z_END = 3499 *# if -1, then the z_end is set as the last z plane*.
      # Proportion of z planes in the substack.
      # Default is 1.6% of the total number of z planes
      > Z_RATIO = 0.016
      > Z_STEP = 1 *# z resolution in micron*
      # Pattern of image in the dataset
      > FILE_PATTERN = “13-17-50_test-hs_UltraII_C{:02d}_xyz-Table Z{:04d}.ome.tif”
  b. Run Step 1.2 of the jupyter notebook “z_projection.ipynb” to define the substacks used for resampling.
  c. Run Step 1.3 of the jupyter notebook “z_projection.ipynb” to save the resampling information into numpy file.
18. Run Step 2 of the jupyter notebook “z_projection.ipynb” to apply Z intensity projection for each substack and save the resampled Z planes. Specify the method for intensity projection in “ZMETHOD”.

**Table 2:** Estimation of error measurement between resampled and non-resampled image stacks

Brain	Substack projected area	Top plane area	Middle plane area	Bottom plane area	Area diff top plane	Area diff middle plane	Area diff bottom plane	Area diff max	Error (%)
Ad male conv 1	2282612	2249173	2248623	2250757	33439	33989	31855	33989	1.489
Ad male conv 2	2177395	2151057	2159248	2167191	26338	18147	10204	26338	1.21
Ad male conv 3	2213684	2126243	2131922	2135662	87441	81762	78022	87441	3.95
Ad male conv 4	2168986	2130573	2125645	2133118	38413	43341	35868	43341	1.998
Ad male GF 1	1620579	1585428	1590926	1605259	35151	29653	15320	35151	2.169
Ad male GF 2	2024436	1989872	1986127	1991852	34564	38309	32584	38309	1.892
Ad male GF 3	1985647	1950899	1971568	1967946	34748	14079	17701	34748	1.75
Ad male GF 4	1865519	1807029	1789458	1792899	58490	76061	72620	76061	4.077
**Mean**	–	–	–	–	–	–	–	–	**2.317**
**SD**	–	–	–	–	–	–	–	–	**1.089**

The brain areas of top, middle and bottom planes of the substack were compared with the subsampled brain area in adult male conventional (conv) and germ-free (GF) samples

# *Choose the z projection method*

> ZMETHOD = ['min','max'] *# must be 'max', 'min', 'avg', 'sum', 'std'*

***Note:*** There are different intensity projections such as minimal, maximal, average and sum intensities. The user chooses a convenient intensity projection for their images. For example, if the ventricles are the bright intensity pixels, then choosing maximal intensity projection is recommended.

***Note:*** Volume measurements after resampling can differ by 2.3 ± 1.1% compared to non-resampled image stacks, indicating that resampling does not substantially affect measurement accuracy (see Table 2).

### Annotation of brain ventricles and brain bounding box position

**Timing: 30 min per brain**

These steps describe how to annotate brain ventricles on the resampled substacks with Arivis Vision4D software and how to position the brain bounding box that will be required to calculate brain and ventricles volume.

***Note:*** Alternative open-source tools can be considered for brain ventricle annotation, although support for large 3D volumetric datasets may be limited.

**CRITICAL:** The protocol was developed with Arivis Vision4D 4.1.2, Wacom tablet Cintiq 22 DTK-2260 and Wacom driver version 6.4.11-1.

19. Import the resampled brain images into Arivis Vision4D by selecting “Import” from the “File” menu.
  a. Navigate to the directory where the user saved the resampled images and choose all of them for importation.
  b. Click “Yes” when asked by the software whether to assume the same structure for all the files.
  c. Click on “Browse” at the “File Name” to determine where to save the Arivis Vision4D project.
  d. Select “Custom import” from the slider to open the “Manual Import Mapper”.
  e. Choose “Pattern matching” from the “Selection” menu.
    ***Note:*** Arivis Vision4D matches the images to the corresponding channels and Z planes automatically.
  f. Click “OK” to let Arivis Vision4D start the importation process.
20. Set the correct pixel size in “View > Show as Info Viewer”.

**Figure 4:**
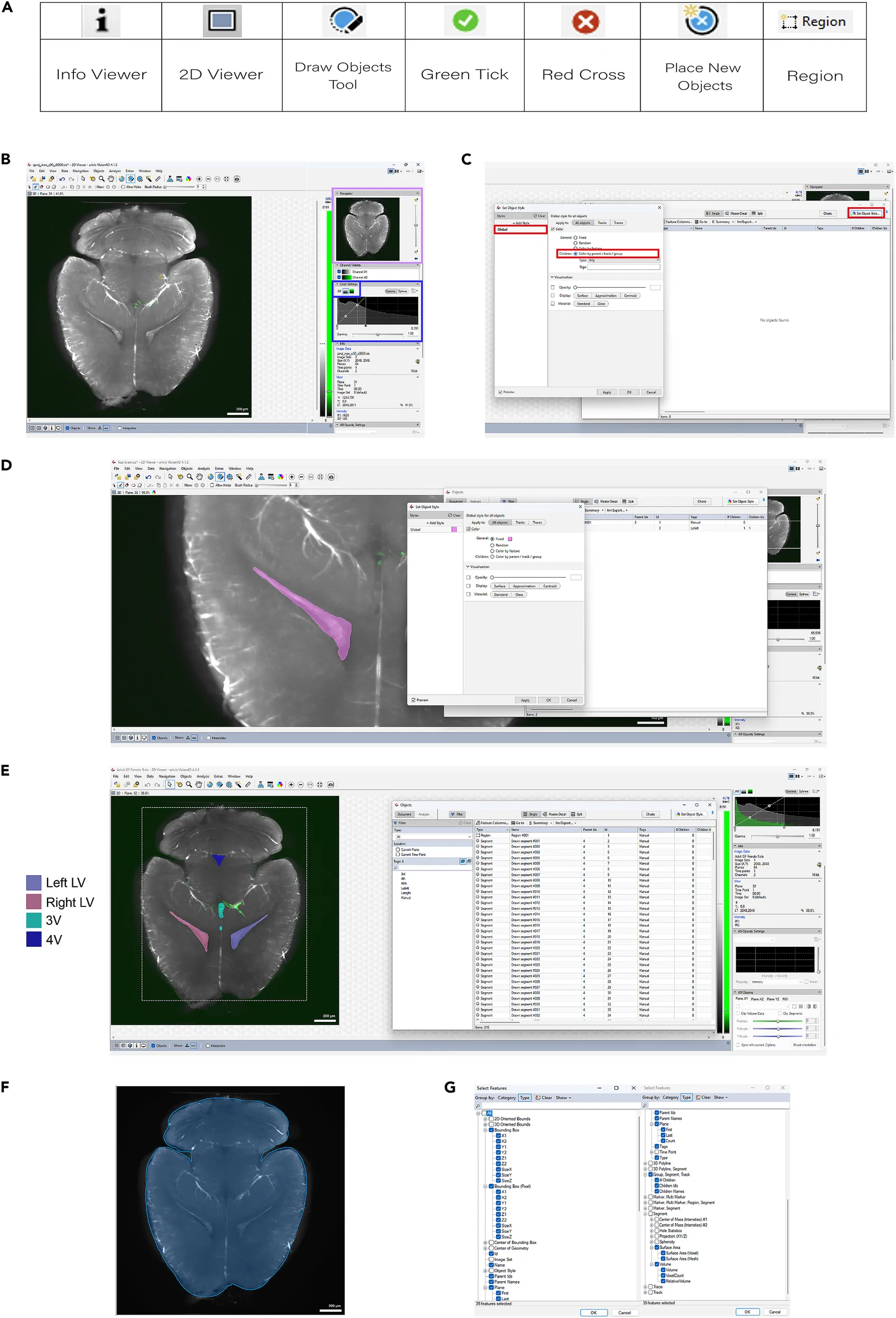
Annotation of brain ventricles, brain bounding box position and annotation of the brain contour (A) Icons used in the Arivis Vision4D software. (B) View of the parameters on the Arivis Vision4D software allowing to change colors settings (blue rectangle) and navigate in the Z dimension (purple rectangle). (C) Parameters on the Arivis Vision4D software to identify each individual structure that will be annotated with a different color (red rectangles). (D) Parameters on the Arivis Vision4D software when annotating ventricles on maximum-intensity projected substack of the imaged brain. (E) Substack of the imaged brain with the four annotated ventricles and position of the bounding box (left), and list of the annotated structures in the substacks (right). (F) Annotation of the brain contour. (G) List of the 35 features that should be selected when exported the list of the annotated structure.

***Note:*** Alternatively, the viewer can be accessed by clicking on the “I” icon (Figure 4A) in the bottom bar of the software.

***Note:*** Having chosen a stepsize of 1 μm in step 14, the value should be 1x1x1.

21. Choose the desired color for each channel
  a. Click in “View > Show Channel Visibility Panel”.
  b. Choose a color for each channel by right clicking in the color squares of the “color settings” menu (Figure 4B, blue rectangle).
  c. Adjust the brightness and contrast by modifying the histogram in the “color settings” menu.

***Note:*** To enhance contrast between the ventricles and the brain parenchyma, it is recommended to set the acquired images with *Lycopersicon esculentum* Lectin DyLight 488 in grey to better visualize the contrast between brain tissue and ventricular space.

22. Before annotating the ventricles, change the “object style” settings to allow the software to identify each individual structure with a different color (Figure 4C, red rectangles to obtain Figure 4E, Methods Video S1). The colors will be defined during the annotation process.
  a. In the “Objects” tab, open the “Object Table”.
  b. Select the “Set Object Style” option and click on “global” in the emergent window (Figure 4C).
  c. In the tab “Apply to: All Objects”, go to the “children” options and select “Color by parent/track/group”.
  d. Click on “Apply” and “Ok” and keep the object table open.
  e. Go back to 2D viewer by clicking in “View > Show 2D viewer” or by clicking in its associated icon (Figure 4A).
23. Connect the Wacom tablet.
24. Proceed with the annotation of the first ventricle. As an example, the next steps detail the annotation of the right lateral ventricle (Figure 4D).
  a. Click on the “Draw Objects Tool” icon (Figure 4A).
  b. Decrease the brush radius to the minimum. Do not activate the option “allow holes”.
  c. On the navigator panel (Figure 4B, purple rectangle), select the first plane in which the right lateral ventricle is visible
  d. Outline its contour.
  e. Click on the green tick to confirm the annotated object and add it to the object table (Figure 4A).
    ***Note:*** The red cross (Figure 4A) can be pressed to eliminate the annotated object before adding it to the object table.
  f. Define a “right lateral ventricle” group in which the following annotations will be added.
    i. Go to the object table and right-click on the annotated object.
    ii. In the groups section, click on “Group Objects”.
    iii. Name your group “right lateral ventricle” and click on “Finish”.
  g. Set a common style to all the annotations in the “right lateral ventricle” group.
    i. Still in the objects table, right click on the generated group and select “Set Object Style”.
    ii. Name your style (e.g., rightLV).
    iii. Select “Fixed” in the general options
    iv. Choose the desired color by clicking on the color box.
      ***Note:*** One color per ventricle is recommended.
    v. Click on “Apply” and “Ok” and go back to the 2D Viewer of the brain.
  h. Annotate the following plane.
    i. On the navigator panel (Figure 4B, purple rectangle), move to the next plane.
    ii. Use the brush to delineate the right lateral ventricle in the new plane.
    iii. Click on the green tick, go to the objects table and right-click on the newly generated object.
    iv. Select “Add to group” and include this new object in the “right lateral ventricle”.
  i. Repeat the step 24-h, plane per plane and dorso-ventrally, until reaching the last plane in which the right lateral ventricle is visible.
    ***Note:*** If all the right lateral ventricle objects are annotated consecutively, the user can add the whole selection to the group after completing the annotation, instead of doing the procedure once per plane.
    ***Note:*** A horizontal mouse brain atlas can be used as a reference to identify the ventricular structures in the different planes.^18^
  j. Once having annotated the right lateral ventricle in all the imaged planes, use the navigator panel to scan the whole brain in Z and verify the annotations.
25. Annotate separately the left lateral, third and fourth ventricles by repeating the process described in step 24 (Figure 4E, Methods Video S1).

***Note:*** Ventricle annotation can be achieved by following either the staining with *Lycopersicon esculentum* Lectin DyLight 488 or TO-PRO-3 Iodide (642/661), or a merge of both. Yet we recommend using the staining with *Lycopersicon esculentum* Lectin DyLight 488 as the primary indicator, as TO-PRO-3 Iodide (642/661) signal lines the border of the ventricles with variable intensity. See troubleshooting 5.

***Note:*** To avoid any potential bias in ventricles annotation among different practitioners, it is recommended that manual annotation is performed by a single practitioner. The ventricle contour used for manual annotation is defined as the location where the pixel intensity corresponds to the average of the intensities of the tissue inside and outside the ventricle.

26. Create the bounding box that will be used to assess total brain volume.
  a. Select a representative plane in the navigator panel.
    ***Note:*** We recommend drawing the bounding box in the same plane throughout the different analyzed brains to ensure reproducibility. This box should be positioned at the mid-sagittal level, where the cross-sectional area is maximal, to ensure that the resulting volume encompasses the full spatial extent of the brain (Figure 4E).
  b. Click on the “Place New Objects” icon and select the region (Figure 4A).
  c. Using the “selection” menu (Figure 4A), draw a rectangle around the brain and use the “edit region” option in the objects menu to adjust its boundaries until they coincide with the brain’s outer limits.
    ***Note:*** No brain tissue should lie outside the rectangle. This box will be used for subsequent brain volume estimation in steps 33-34.
27. Save the Arivis Vision4D file and, in the objects table, select “Excel export” to generate an excel file and proceed to quantification of the brain and brain ventricle volume. Specify:
  a. Objects: Select “All”.
  b. Report: Select “Single table”
  c. Features: 35 features should be selected (Figure 4G).

**Figure 5:**
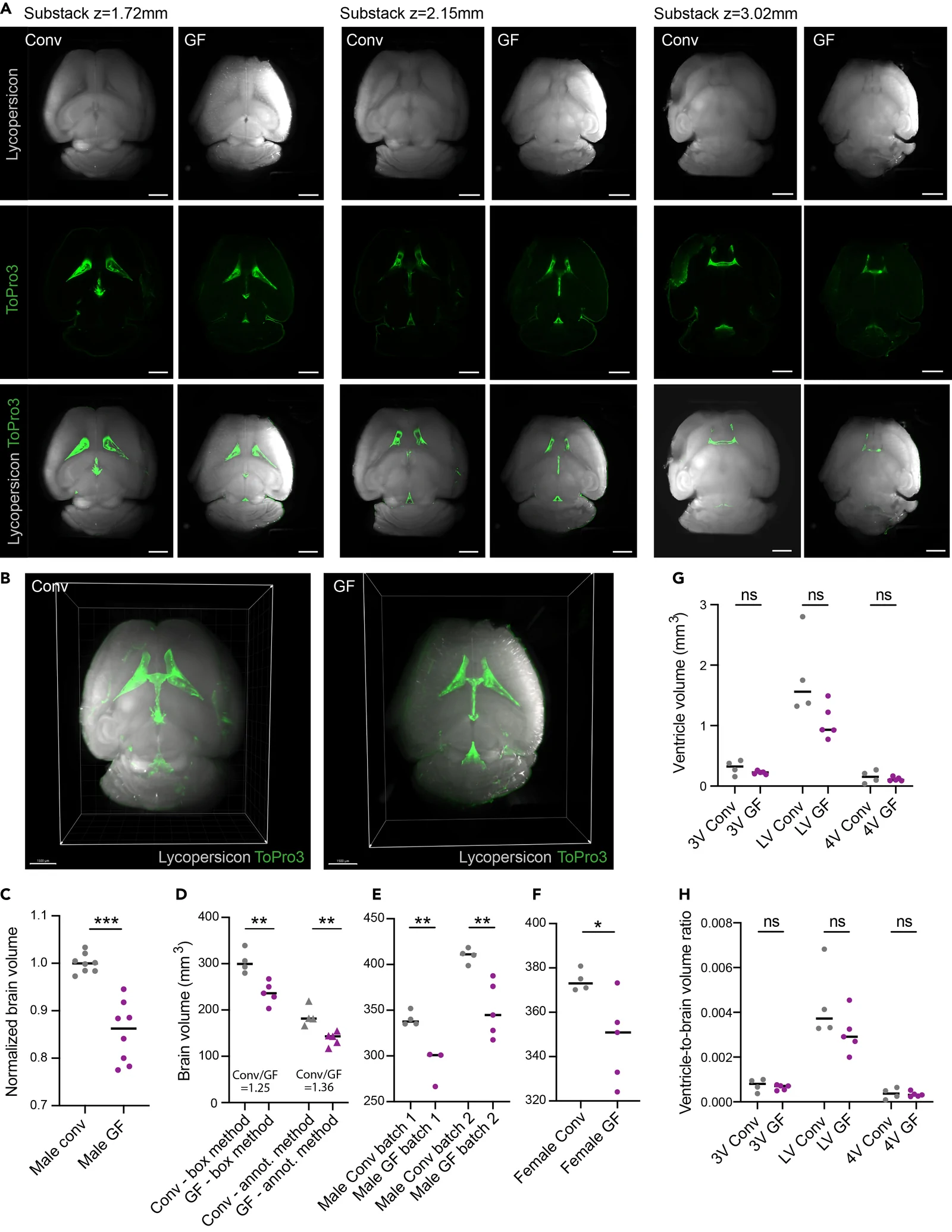
Validation of the method showing a reduction in brain volume in adult germ-free mice (A) Detection of conventional and germ-free adult brain tissues stained with the *Lycopersicon esculentum* Lectin DyLight 488 (grey) and TO-PRO-3 Iodide (642/661) (green), in the various maximum-intensity-projected-substacks, along the Z dimension. Bar: 1.5mm. (B) Three-dimensional rendering of the brains imaged in (A). Bar: 1.5mm. (C) Normalized brain volumes of conventional and germ-free adult male mice. (D) Brain volumes of conventional and germ-free adult male mice calculated using either the brain bounding box method (left) or the annotation of the brain contour method (right). (E) Brain volume of conventional and germ-free adult male mice from two different experiments (batch 1 and 2). (F) Brain volume of conventional and germ-free adult female mice. (G and H) Third (3V), lateral (LV) and fourth (4V) ventricles volumes of conventional and germ-free adult male mice (batch 2), in absolute values (G) or normalized over their respective brain volume (H). Conv: conventional, GF: germ-free. Statistical comparison in (C–H): unpaired *t* test; bar represented: median; ∗p < 0.05; ∗∗p < 0.01; ∗∗∗p < 0.001; p > 0.05 is nonsignificant (ns).

***Optional:*** Brain volume can also be calculated by annotating the brain outline on each projected image substack, like for the different ventricles (see Figures 4F and 5D in expected outcomes). If this option is chosen, create a new Arivis Vision4D file dedicated to brain annotation and repeat the steps 19-27; however, instead of delineating the ventricles, delineate the brain contour in every plane of the resampled stack. Alternatively, the brain contour can be only annotated in one out of every ten substacks; next, the cross-sectional areas corresponding to the missing Z planes can be interpolated from the areas of the two neighboring Z planes above and below of the missing Z plane (see steps 33-34).

Methods Video S1. Visualization of the four annotated ventricles and the bounding box in the various maximum-intensity-projected-substacks, along the Z dimension, related to steps 22 and 25

### Brain and brain ventricle volume measurements

**Timing: 5 min per brain**

These steps describe how to calculate the brain and ventricle volume based on the annotations and brain bounding box generated on Arivis Vision4D.

**CRITICAL:** Calculation of the ventricles and brain volume uses the Python code provided in the jupyter notebook “measure_ventricle_volume.ipynb”. The jupyter notebook is found at https://gitlab.pasteur.fr/iah-public/ventricle-volume-measurement. Additionally, the code requires 2 files as an input: the excel file obtained in step 27 and the numpy file containing the resampling information, generated in step 17c.

28. Import the data containing the brain bounding box and ventricles annotation by running the jupyter notebook “measure_ventricle_volume.ipynb”.
  a. Run Step 1.1 of the jupyter notebook “measure_ventricle_volume.ipynb” to import the Excel file that contains the annotations of ventricles and the brain bounding box from Arivis Vision4D. Specify the file path in “TBL_FILE.”
    # Excel file from Arivis
    > TBL_FILE = “…/Segmented brains/Adult GF Male 1 – done/Arivis files/Adult GF Male 1-features.xlsx”
    **CRITICAL:** In the “Type column” of the Excel file, the ventricle annotations must be set to “Segment”, and the bounding box must be set to “Region”.
  b. Only if the user has drawn the brain outline in every plane (see note in step 27), run step 1.2 of the jupyter notebook “measure_ventricle_volume.ipynb” to import a third input file, the Excel containing the brain annotations from Arivis Vision4D. Specify the file path in “TBL_BRAIN_FILE”. Otherwise, omit this step.
    # *Excel file containing brain annotation*
    > TBL_BRAIN_FILE = “…/Segmented brains/Adult GF Male 1 – done/Brain volume/BV_Adult GF Male 1-features.xlsx”
    **CRITICAL:** In the “Type” column of the Excel file, the brain annotations must be set to “Segment”.
29. Run Step 2 of the jupyter notebook “measure_ventricle_volume.ipynb” to set the voxel size value (in microns).

> dz = 1 # micron

> dx = dy = 5.16 # micron

***Note:*** The voxel size is determined by the light-sheet microscope configuration and the parameters used during the acquisition. This value can be obtained by accessing the metadata of the Light Sheet dataset, e.g. opening the images in Fiji/Image J software, that should be stored in the first acquired image.

30. Run Step 3 of the jupyter notebook “measure_ventricle_volume.ipynb” to import the resampling information obtained from running the notebook “z_projection.ipynb”. Specify the file path in “INFO_FILE”.

# *Path to the sampling info*

# Use '/' for Linux or Mac system, '\\' for Windows

> INFO_FILE = "../../...npy"

31. Run Step 4.1 of the jupyter notebook “measure_ventricle_volume.ipynb” to compute the ventricle volume of each substack.
32. Run Step 4.2 of the jupyter notebook “measure_ventricle_volume.ipynb” to compute the total ventricle volume of the whole image stack.
33. Normalize the ventricle volume by:
  a. Total number of Z planes used for image stack resampling (see step 17a, iv) by running the step 5.1 of the jupyter notebook “Measure_ventricle_volume.ipynb”.
  b. Total brain volume, either measured by the bounding box volume method (see step 26) or by the annotated brain contour method (see note in step 27). For such normalizations, run the step 5.2 or 5.3 of the jupyter notebook “Measure_ventricle_volume.ipynb”, respectively.

***Note:*** When the total height of the brain exceeds the working distance of the microscope, the brain volume can only be quantified with the bounding box volume method, by replacing the “info [‘nz’]” parameter in 5.2 [13] by the value of the total height of the brain recorded in step 16 (see troubleshooting 4).

*# Bounding box volume*

*>* bbox_volume = bbox_sizex ∗ bbox_sizey ∗ info['nz'] ∗ dx ∗ dy ∗ dz

> print(“Bounding box volume (micron):”,bbox_volume)

34. For brain volume quantification, use the values generated in Step 5.2. [13] or 5.3 [16] of the jupyter notebook “Measure_ventricle_volume.ipynb”, depending on the method used.

## Expected outcomes

Changes in brain and brain ventricular volume are increasingly recognized as early markers of neurodevelopmental and neurodegenerative disease and have recently been associated with microbiome dysbiosis.^6^^,^^9^^,^^10^^,^^19^ Such brain alterations can be assessed by using our protocol, which can provide qualitative and quantitative evidence of brain and brain ventricles volume at different stages of development and disease progression.

We first tested our method by comparing the brains of adult male germ-free mice, which are devoid of microbiota, to their conventional counterparts with normal microbiota. Thompson et al. recently used MRI to describe reduced brain volume in germ-free mice when compared to conventional animals, reporting greater differences in males than in females.^6^ In our workflow, resampling of the imaged brains after clearing allows for the detection of brain tissue stained with the *Lycopersicon esculentum* Lectin DyLight 488, in various maximum-intensity-projected-substacks along the Z axis (Figure 5A). While the ventricles appear to be unstained by *Lycopersicon esculentum* Lectin DyLight 488, their outline can be detected with TO-PRO-3 Iodide (642/661) signal, which is particularly evident in three-dimensional reconstructions of the acquired brain images using Imaris Viewer (Figure 5B). Following brain volume quantification normalized by the bounding box, male germ-free brains were found to exhibit reduced brain volume when compared to their conventional counterparts (Figure 5C), in agreement with the findings of Thompson et al.^6^ This difference in brain volume between germ-free and conventional adult male mice can be detected by using both the brain bounding box method and the annotation of brain contour method (see step 27), but higher brain volume values are observed in the first case, likely because the bounding box is positioned where the brain is largest (Figure 5D). However, while the difference in brain volume between germ-free and conventional adult male mice is reproducible from one experiment to another, the values vary between different experiments, likely due to a batch effect induced during the dehydration process (see troubleshooting 6) (Figure 5E). Thompson et al. additionally described a reduction in female germ-free brain volume, to a lesser extent than in males, which can also be captured with the proposed workflow (Figure 5F). Finally, no difference in volume is observed between ventricles in conventional and germ-free males when the volume of the different ventricles is normalized to brain volume (Figure 5H).

To validate our method in a context of brain development, we used a transgenic mouse model, Nestin::Cre;Ift88^fl/ko^, in which radial glial cells (Nestin-positive) lose *Ift88*, preventing primary cilia formation and leading to a dilation of the brain ventricles during late embryonic stages (ventriculomegaly). Ultimately, such dilation causes hydrocephalus during postnatal stages.^7^ As expected, the brain volumes of male and female postnatal day 8 (P8) control mice (*Nestin::Cre;Ift88*^*fl/+*^), measured with the bounding box method, were lower than those observed in adults (Figure 6A). While the brain volume of hydrocephalic P8 mice (*Nestin::Cre;Ift88*^*fl/k*o^) was equivalent to that of control P8 mice (Figures 6A and 6B), the lateral ventricle volume of P8 hydrocephalus mice was approximately ten times greater than that of the control P8 mice (Figures 6B–6D). This observation is consistent with the enlargement of the lateral ventricles in mice with hydrocephalus measured in Foerster et al., although it was quantified on two-dimensional images and expressed in surface area units.^7^

**Figure 6:**
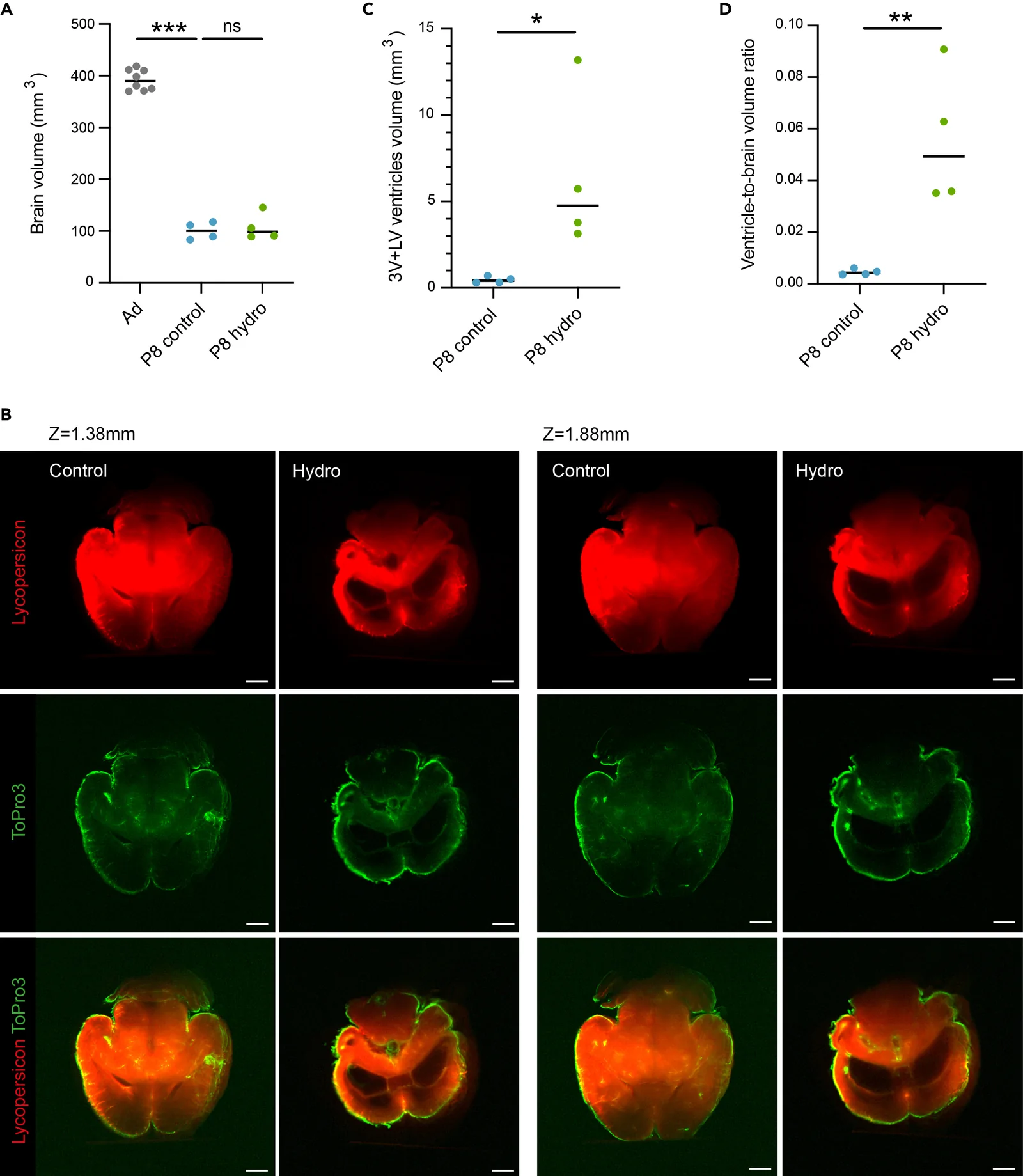
Validation of the method showing an enlargement of the ventricles in P8 brains of mice with hydrocephalus (A) Brain volume of female and male adult, P8 control (*Nestin::Cre;Ift88*^*fl/+*^) and P8 with hydrocephalus mice (*Nestin::Cre;Ift88*^*ko/fl*^). (B) Sections at z=1380 and z=1880μm of P8 control and P8 with hydrocephalus imaged brains after clearing and staining with *Lycopersicon esculentum* Lectin DyLight 488 (red) and TO-PRO-3 Iodide (642/661) (green). Bar: 700μm. (C) Third and lateral ventricles volume of P8 control and P8 with hydrocephalus mice. (D) Third and lateral ventricles volume in (C) normalized over the respective brain volume. Hydro: hydrocephalus. Statistical comparison in (A): one-way ANOVA with Tukey’s multiple comparison, in (C-D): unpaired *t* test; bar represented: median; ∗p < 0.05; ∗∗p < 0.01; ∗∗∗p < 0.001; p > 0.05 is nonsignificant (ns).

Overall, the proposed workflow allows the visualization and precise measurement of brain and ventricles volumes in both adult and neonatal mice, producing reliable datasets that align with findings reported in the literature.

## Limitations

Annotation of brain ventricles using this workflow involves several inherent trade-offs that should be considered when adapting it to other applications. FDISCO clearing protocol was chosen to prioritize short processing time and robust fluorescence preservation over optical transparency, which in certain cases resulted in suboptimal clarity of the tissue. However, since the brain ventricles show a great contrast with the tissue parenchyma, and the *Lycopersicon esculentum* Lectin DyLight 488 dye is robust in staining the vasculature, the achieved clarity was generally sufficient for the intended analysis. Alternatively, the workflow is also compatible with brains cleared using other clearing protocols.

In addition, the dehydration induced during tissue clearing induces tissue shrinkage that can vary between processing batches, hindering quantitative comparisons when the samples have not been processed in parallel. Also, if experimental groups differ in tissue water content (e.g., due to edema, injury, or inflammation), the dehydration process could differentially affect tissue shrinkage and may influence volumetric measurements. Nevertheless, the workflow allows the parallel processing of large numbers of samples within the same batch, and batch-dependent effects can be addressed by appropriate batch-normalization approaches.

Finally, this light-sheet microscopy-based protocol imposes limitations on the number of anatomical structures that can be analyzed simultaneously. Such restriction is a result of the number of available fluorescence channels in the microscope and the requirement for fluorescent markers to be capable of fully penetrating brain tissue while providing specific staining patterns with low background staining. Consequently, this protocol is best suited for the targeted analysis of selectively labeled structures rather than unbiased visualization of all brain regions.

## Troubleshooting

### Problem 1

Brains are not totally cleared after incubation in DBE and look opaque (step 7c).

Possible reasons are 1) Incomplete dehydration, 2) Incomplete delipidation 3) Oxidation of THF or Dichloromethane solution, and 4) Plastic incompatibility.

### Potential solution

Solution 1: Use dehydration solutions that are freshly prepared (step 5).

Solution 2: Use delipidation solution that is freshly prepared and use a new tube. If brains in DBE are not sufficiently cleared, immerse the brain again in 100% THF for 12h and re-do the delipidation and clearing steps (steps 6 and 7).

Solution 3: Make sure that the THF is complemented with BHT and Dichloromethane with amylene as stabilizers, preventing the accumulation of peroxide byproducts that affect clarification. During THF and Dichloromethane treatment, the tissue should be processed in a container filled with a minimum of air to prevent oxidation.

Solution 4: Use recommended glass or polypropylene tubes and not polystyrene tubes, since they can react with the clearing reagents

### Problem 2

Brain is not stable in the holder (step 10) and moves during light sheet setup and/or acquisition (steps 11 to 16).

Possible reasons are 1) the sample is not sufficiently fixed in the holder or 2) the light sheet objective contacts the brain because the working distance is exceeded.

### Potential solution

Solution 1: Tighten the brain on the sample holder (step 10) or replace the holder (see step 9c) and reimage the sample. Ensure that the tissue is sufficiently immobilized to prevent displacement while avoiding excessive force that may cause mechanical damage. If the sample detaches from the holder and falls into the DBE cuvette, immediately switch off the laser before retrieving the tissue. Be mindful not to cause DBE spillage that can damage the microscope.

Solution 2: Before immersing the objective, adjust the sample position in the Z direction so that the light sheet illuminates the top of the tissue to prevent the objective from crashing with the sample while lowering it (step 11). While setting up the scan range in Z dimension, select “Set at Zero” in the “x-y-z Table Z/Current position” section while selecting the “Start” position to prevent exceeding the microscope working distance while selecting the “End” position (step 13).

### Problem 3

Illumination of the brain is not homogeneous; the image is blurred or shadowed (step 12g).

Possible reasons: 1) the illumination of the brain is not homogenous along the Y dimension, 2) the illumination is not homogeneous along the X dimension, 3) the imaging is out-of-focus, 4) choice of incorrect clearing solution, 5) the imaging cuvette is not properly filled, 6) the tissue is damaged and 7) there is an air bubble in the DBE cuvette.

### Potential solution

Solution 1: Adjust the sheet width of the light to illuminate homogeneously the surface of the brain (step 12g).

Solution 2: Choose dual-sided illumination to detect the signal homogeneously on both sides of the brain (step 12g). Note that the acquisition time will be increased.

Solution 3: Zoom digitally during imaging setup focusing on a thin structure and adjust the focus (step 12-e). Be careful not to excessively immerse the objective while adjusting the focus to avoid contacting the sample (step 12-d).

Solution 4: Select the correct imaging liquid (DBE) in “Ultrall/Measurement/Sample/Liquid”.

Solution 5: Fill the imaging cuvette with DBE (step 8).

Solution 6: Be careful while harvesting, transferring from one solution to another (steps 4 to 7) and mounting the brains in the imaging holder (step 10).

Solution 7: Move the objective out of the cuvette and if the air bubble remains, try to remove it with forceps.

### Problem 4

The brain cannot be imaged in its entirety in Z dimension (step 13).

Possible reason: the height of the brain exceeds the microscope working distance.

### Potential solution

Solution: Position the brain in the imaging holder to allow the acquisition of the area of interest (at the top). After the acquisition, slightly move up the objective while still immersed in DBE and reselect the “Start” and “End” positions in Z dimension, even if the sample looks blurry, to obtain the full height of the brain (step 16) that the user will need for final brain volume calculation in steps 33 and 34.

### Problem 5

TO-PRO-3 Iodide (642/661) marker is lining the ventricles with different intensity (step 12) which hinders ventricle annotation (steps 24 and 25).

Possible reason: incomplete marker penetration.

### Potential solution

Solution: Removing meninges can improve fluorescent markers penetration.

### Problem 6

Batch effect in calculation of brain volumes (expected outcomes, Figure 5E and limitations).

Possible reason: clearing process and tissue shrinkage after dehydration vary from experiment to experiment.

### Potential solution

Solution: When multiple samples need to be compared, all samples must be clarified in parallel and at the same time. Alternatively, batch normalization needs to be performed when comparing samples processed in different batches.

## Resource availability

### Lead contact

Further requests for resources should be directed to and will be fulfilled by the lead contact, Laetitia Travier (laetitia.travier@pasteur.fr).

### Technical contact

Further information should be directed to and will be fulfilled by the technical contact, Ana Blas-Medina (ana.blas-medina@pasteur.fr).

### Materials availability

This study did not generate any new unique reagent.

### Data and code availability

• The accession number for the images analyzed in Figures 5 and 6 reported in this paper is BioImage Archive: S-BIAD2844 (https://www.ebi.ac.uk/biostudies/bioimages/studies/S-BIAD2844?key=adece213-8829-470b-a8a0-1c0628203e86).
• Original codes supporting the current study have been deposited at: https://gitlab.pasteur.fr/iah-public/ventricle-volume-measurement.

## Acknowledgments

The research in Deczkowska’s lab is supported by the G5 funding from 10.13039/501100003762Institut Pasteur, the European Research Council Starting grant (BrainGate), 10.13039/501100001665French National Research Agency (DevoCP ANR-22-CE14-0007), Ville de Paris EMERGENCE(S) grant, Alzheimer’s Association Research Grant (AARG-22-917964), 10.13039/501100006424Fédération pour la Recherche sur le Cerveau, ERA-Net NEURON 2024, and Don Explore AD (Programme Explore de l’Institut Pasteur). Our lab is part of the DIM C-BRAINS, funded by the Conseil Régional d’Île-de-France. We thank all members of the Deczkowska lab, past and present, for technical support and fruitful discussions. We sincerely appreciate the support of the Image Analysis Hub in Institut Pasteur and the France-BioImaging infrastructure (https://ror.org/01y7vt929) supported by the 10.13039/501100001665French National Research Agency (ANR-24-INBS-0005 FBI BIOGEN). We gratefully acknowledge the UTechS Photonic BioImaging (Imagopole), C2RT, Institut Pasteur, supported by the French National Research Agency (France BioImaging, ANR-24- INBS- 0005 FBI (BIOGEN); Investments for the Future). We acknowledge Institut Pasteur for making the UltraMicroscopeII microscope available for this work and the Paris Brain Institute ICM-Quant platform for their technical support. We thank the Central Animal Facility of Institut Pasteur for taking care of the animals used in this study. The Spassky lab is funded by the 10.13039/501100001665Agence Nationale de la Recherche (ANR-22-CE16-0011 and ANR-21-CE16-0016), the 10.13039/501100002915Fondation pour la Recherche Médicale (FRM) (EQU FRM 202103012767), and the European Research Council (ERC consolidator grant 647466). The project leading to these results has received funding from “la Caixa” Foundation (ID 100010434), under agreement <LCF/BQ/EU23/12010114>. We thank Jean-Léon Thomas for fruitful discussions. The graphical abstract was created with BioRender. This research was funded, in whole or in part, by the French National Research Agency (DevoCP) and ERC (StG BRAINGATE). For the purpose of open access, the author has applied a CC-BY public copyright license to any Author Manuscript version arising from this submission.

## Author contributions

Conceptualization and methodology, A.B.-M., M.S.P., S.A.-M., J.-Y.T., J.F., A.D., and L.T.; investigation and data analysis, A.B.-M., M.S.P., S.A.-M., and L.T.; hydrocephalus mouse model, N.S.; writing, A.B.-M., M.S.P., and L.T.; funding acquisition, A.D.

## Declaration of interests

The authors declare no competing interests.
